# Evaluating the impact of partial volume correction on FDG PET radiomics stability in lymphoma lesions

**DOI:** 10.1007/s13246-026-01717-3

**Published:** 2026-03-09

**Authors:** Setareh Hasanabadi, Mohammad Saber Azimi, Mehrdad Bakhshayesh Karam, Hossein Arabi

**Affiliations:** 1 Department of Medical Radiation Engineering, Shahid Beheshti University, Tehran, Iran; 2 Doctoral School of Applied Informatics and Applied Mathematics, Óbuda University, Budapest, Hungary; 3 Nuclear Medicine Department, Masih Daneshvari Hospital, Tehran, Iran; 4 Division of Nuclear Medicine and Molecular Imaging, Geneva University Hospital, Geneva, 1211 Switzerland

**Keywords:** ^18^F-FDG PET, lymphoma, radiomics, Stability, partial volume correction (PVC), lesion volume

## Abstract

**Purpose:**

High Stability of radiomic features is critical for developing reliable imaging biomarkers that can support risk stratification, treatment response assessment, and personalized therapy in lymphoma patients. To evaluate how partial volume correction (PVC) affects the Stability of ^18^F-FDG PET radiomic features in lymphoma lesions, with respect to lesion volume and tissue type.

**Methods:**

This single-center retrospective study included 131 newly diagnosed lymphoma patients (2014–2024) who underwent baseline ^18^F-FDG PET/CT. In total, 1,603 lesions (1,302 lymph nodes, 117 spleen/liver, 150 bone, and 34 bone and soft-tissue) were semi-automatically segmented and grouped by volume (< 3, 3–10, 10–30, > 30 mL) and tissue type. Ninety-three radiomic features were extracted from non-PVC and PVC images processed with the Richardson–Lucy (RL) and Reblurred Van Cittert (RVC) algorithms after isotropic resampling (3 mm) and discretization (0.25 SUV bin size), following IBSI guidelines. Stability was quantified using the coefficient of variation (CoV) and the intraclass correlation coefficient (ICC_2_, absolute agreement), with statistical comparisons performed via Mann–Whitney U tests and false discovery rate (FDR) correction.

**Results:**

PVC significantly improved feature Stability, particularly for large lesions (> 30 mL), with median ICC_2_ > 0.90 across most feature categories (e.g., First-Order = 0.99, GLSZM = 0.97, NGTDM = 0.97). Small lesions (< 3 mL) showed lower stability (ICC_2_ = 0.84–0.94) and higher CoV (0.09–0.21), mainly in texture-based features. First-Order and GLCM features were the most robust overall (ICC_2_ = 0.92–0.99; CoV = 0.07–0.11). Bone and spleen lesions exhibited the highest Stability (median ICC_2_ ≈ 0.95), whereas lymph node and liver features were more variable. All volume- and tissue-dependent differences remained significant after FDR correction (*p* < 0.05).

**Conclusion:**

PVC using RL and RVC markedly enhances FDG-PET radiomic Stability in lymphoma, particularly for larger and structurally uniform lesions. Robust features such as First-Order and GLCM can support standardized radiomics workflows and the development of reliable biomarkers for prognosis and personalized therapy. Additionally, PVC reduces variability in texture features, especially in small or heterogeneous lesions. Multicenter validation would further strengthen generalizability beyond this single-center setting.

**Supplementary Information:**

The online version contains supplementary material available at https://doi.org/10.1007/s13246-026-01717-3.

## Introduction

^18^F-Fluorodeoxyglucose positron emission tomography (^18^F-FDG PET) plays a central role in lymphoma management, supporting diagnosis, staging, treatment planning, and therapy response assessment [1–3]. By quantifying tumor metabolism, ^18^F-FDG PET provides valuable insights into disease burden and heterogeneity, which are essential for personalized treatment strategies in both Hodgkin and non-Hodgkin lymphoma [4, 5].

Radiomics, the high-throughput extraction of quantitative features from medical images, has expanded the diagnostic and prognostic capabilities of PET. Radiomic features describing tumor intensity, texture, and shape have demonstrated prognostic value for outcomes such as progression-free and overall survival and may complement, or in some settings outperform, traditional clinical indices such as the International Prognostic Index (IPI) [6–10]. However, the clinical translation of radiomics remains limited by poor feature Stability, which undermines the stability of predictive models across scanners and imaging centers.

One major source of variability arises from partial volume effects (PVE), caused by the limited spatial resolution of PET systems (typically 4–6 mm full width at half maximum (FWHM)) [11]. PVE leads to signal spill-over and underestimation of tracer uptake, particularly in small (< 3 mL) or heterogeneous lesions, which can in turn affect radiomic feature reliability [12, 13]. These artifacts distort both intensity- and texture-based metrics, compromising quantitative reliability.

A growing body of recent studies has demonstrated the utility of partial volume correction (PVC) techniques, including classical iterative methods such as Richardson–Lucy (RL) and Reblurred Van Cittert (RVC), as well as emerging learning-based approaches, for mitigating partial volume effects and improving quantitative accuracy in molecular imaging [14–16]. Quantitative PET studies in solid tumors such as non-small-cell lung cancer (NSCLC) have demonstrated good repeatability and stability of metabolic and textural parameters, particularly in larger lesions [17]. However, the impact of partial volume correction (PVC) on radiomic stability in lymphoma, where both nodal and extranodal sites exhibit high biological and anatomical heterogeneity, remains largely unexplored.

In oncological PET, systematic reviews and recent radiomics studies have reported that PVC can improve quantitative accuracy and, in some settings, enhance the robustness of derived imaging features [18, 19] .

Lymphoma presents unique challenges for radiomics due to its multi-organ involvement and broad lesion-size distribution. Across cancer imaging, both anatomical site and underlying tissue heterogeneity can influence the metabolic and textural characteristics of lesions and, consequently, radiomic feature stability; this is particularly relevant in lymphoma where lesions can arise in diverse nodal and extranodal compartments (e.g., spleen, liver, and bone) [20, 21]. PET radiomic robustness has been reported in a limited number of studies [22–25], with only a few assessing the impact of PVC. While the impact of PVC on radiomic robustness has been explored in other malignancies [18], no prior study has systematically evaluated PVC’s effect on FDG PET radiomics robustness in lymphoma, particularly considering the combined influence of lesion volume and multi-organ tissue heterogeneity. sites remain largely unexplored.

The present study provides the first comprehensive evaluation of ^18^F-FDG PET radiomic robustness following PVC in lymphoma, encompassing both nodal and extranodal lesions. By quantifying how PVC (via RL and RVC) affects feature stability across lesion sizes and tissue categories, this work delivers crucial evidence for developing standardized radiomic pipelines. The findings aim to identify robust and generalizable features suitable for multicenter studies, advancing reproducible imaging biomarkers for personalized therapy in lymphoma.

## Material and method

### Data acquisition

This study included patients who underwent ^18^F-FDG PET/CT at Masih Daneshvari Hospital (Tehran, Iran) between 2014 and 2024. All procedures complied with the Declaration of Helsinki .The study protocol was approved by the Medical Ethics Committee of Shahid Beheshti University of Medical Sciences (approval code: IR.SBMU.NRITLD.REC.1402.060). Because this was a non-interventional imaging analysis using anonymized data, the requirement for informed consent was waived.

Eligible participants were newly diagnosed lymphoma patients who underwent baseline ^18^F-FDG PET/CT for staging and whose diagnosis was confirmed by histopathology. Exclusion criteria were as follows: (1) non-original or incomplete PET/CT datasets, (2) negative or indeterminate findings, (3) suspected infections or inflammatory conditions, (4) known hepatic fibrosis or cirrhosis affecting physiological liver uptake, (5) concurrent or recent malignancies (e.g., breast cancer), and (6) scans reconstructed using non-standard protocols.

### Imaging protocol

PET/CT imaging was performed on a GE Discovery 690 scanner (GE Healthcare, Milwaukee, WI, USA) equipped with time-of-flight (TOF) capability and a 64-slice CT system. Whole-body acquisitions were obtained from the vertex to mid-thigh. Image reconstruction was performed using the Vue Point HD Sharp (VPHDS) iterative algorithm provided by the manufacturer.

All patients fasted for at least 6 h before tracer administration, ensuring a blood glucose level < 140 mg/dL at the time of injection. A mean interval of 60 ± 10 min (range, 45–75 min) between tracer injection and image acquisition was maintained. Each PET bed position was acquired for 2–3 min. The PET slice thickness was 3.75 mm, while low-dose CT was acquired with 1.33–2.5 mm slice thickness. CT parameters included a tube voltage of 120 kVp, tube current modulation between 50 and 150 mA (automatically adjusted to patient body habitus), and a helical pitch of 0.9. PET data were corrected for scatter, randoms, attenuation (using CT-based attenuation correction), and decay before reconstruction.

All PET/CT imaging was performed on a GE Discovery 690 scanner using the Vue Point HD Sharp (VPHDS) iterative reconstruction algorithm with 3 iterations, 18 subsets, and a 6.4-mm Gaussian post-reconstruction filter. Reconstruction parameters were uniform across all included patients. Scans reconstructed with alternative protocols (e.g., Vue Point FX Sharp [VPFXS]) were excluded during initial data curation (*n* = 26), yielding the final cohort of 131 patients.

### Image evaluation and lesion segmentation

**Fig. 1:**
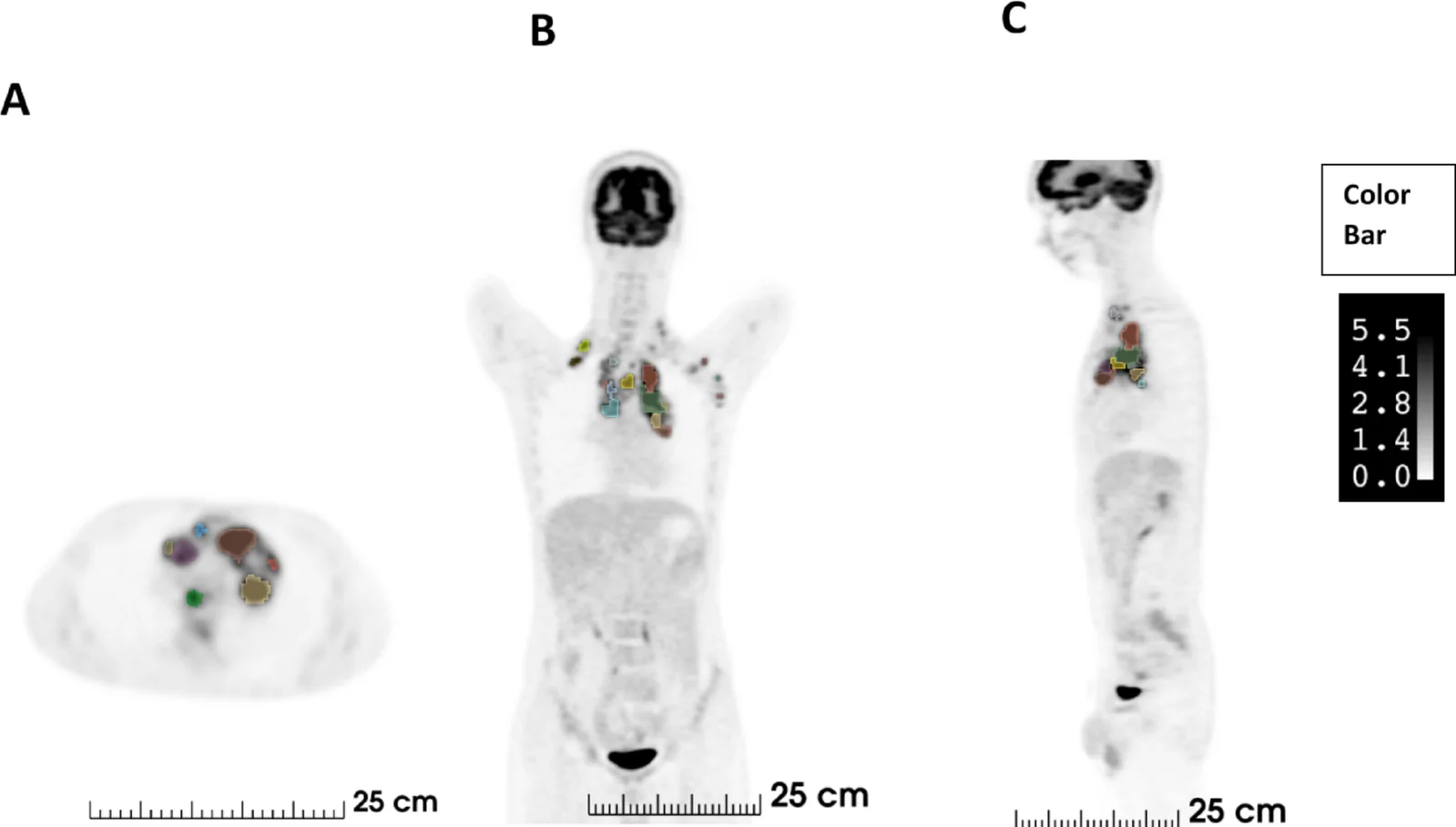
Representative example of the semi-automatic graph-based segmentation method (**A** transaxial, **B** coronal, and **C** sagittal view)

All PET/CT datasets were reviewed by a board-certified nuclear medicine physician with over ten years of experience and a board-certified radiologist with more than 34 years of experience. The primary evaluation involved disease staging and identification of both nodal and extranodal disease sites. For each patient, all metabolically active lesions were considered, including both nodal and extranodal sites (e.g., spleen, liver, bone, and soft tissue), with no numerical limit on the number of lesions per patient (Fig. 1).

Lesion segmentation was performed using the PET Tumor Segmentation effect within the Editor module, a semi-automated gradient-based method [26], implemented in the 3D Slicer platform (version 5.6.0) [27].

The nuclear medicine physician visually inspected and manually refined each delineation to ensure anatomical and metabolic accuracy. A second reader independently verified a subset of the segmentations for quality control.

### Image preprocessing and feature extraction

PET images were resampled to an isotropic voxel size of 3 × 3 × 3 mm³ to ensure rotational and inter-scanner consistency. All PET images were converted to standardized uptake value (SUV) maps normalized to body weight (SUVbw) and discretized using a fixed bin width of 0.25 SUV, following the Image Biomarker Standardization Initiative (IBSI) guidelines [28]. Radiomic features were extracted using the PyRadiomics package (version 3.0.1) [29] integrated within 3D Slicer [27]. Feature extraction was performed separately for each lesion on both non–PVC and PVC-corrected PET images (processed with the RL and RVC algorithms). A total of 93 radiomic features were computed and categorized into six groups:

- First-Order Statistics (18 features).
- Gray-Level Co-occurrence Matrix (GLCM, 24 features).
- Gray-Level Run Length Matrix (GLRLM, 16 features).
- Gray-Level Dependence Matrix (GLDM, 14 features).
- Gray-Level Size Zone Matrix (GLSZM, 16 features).
- Neighborhood Gray Tone Difference Matrix (NGTDM, 5 features).

All features were extracted in accordance with IBSI definitions to ensure cross-platform comparability.

### Partial volume correction (PVC)

Partial volume correction was performed using the PETPVC toolbox (version 1.2.0; University College London, London, UK) [14], a C++-based software package built on the Insight Segmentation and Registration Toolkit (ITK) framework. This toolbox implements several established post-reconstruction PVC algorithms. In this study, two widely used deconvolution-based methods, the Richardson–Lucy (RL) and Reblurred Van Cittert (RVC) algorithms, were applied to all reconstructed PET images.

Both RL and RVC are voxel-wise, mask-independent iterative deconvolution approaches that use knowledge of the imaging system’s point spread function (PSF) to iteratively compensate for partial volume effects (PVE). The algorithms operate in the image domain and do not require anatomical segmentation masks. Each iteration estimates a corrected image by convolving the previous estimate with a 3D Gaussian kernel representing the PSF, followed by a normalization step to prevent noise amplification. In this study, 10 iterations were applied for RL and 8 iterations for RVC. These values were selected based on prior validation studies demonstrating that activity recovery converges within this iteration range, while further iterations lead to increased noise amplification in clinical PET images [30].

For the Discovery 690 PET/CT scanner (GE Healthcare, Milwaukee, WI, USA) used in this study, equipped with time-of-flight (TOF) and PSF modeling via Vue Point HD Sharp (VPHDS) reconstruction, the intrinsic system spatial resolution was characterized by a three-dimensional Gaussian PSF with a FWHM of approximately 4.3 mm in the transaxial (x, y) directions and 4.8–5.0 mm in the axial (z) direction [31]. These parameters were incorporated into PETPVC as the Gaussian kernel under a shift-invariant assumption for both RL and RVC deconvolution procedures.

### Statistical analysis

Radiomic features were extracted from all segmented lesions across nodal (lymph node) and extranodal regions (spleen, liver, bone, and soft tissue). Lesions were further stratified into four volume groups: <3 mL, 3–10 mL, 10–30 mL, and > 30 mL. Shape-based descriptors (e.g., mesh volume, surface area) were excluded from stability analysis to focus on intensity- and texture-derived features. Non-numeric or missing values were removed prior to computation.

In this study, radiomic feature stability was defined as the degree to which feature values remain consistent across non-PVC and PVC-corrected PET images. Stability was quantified using the intraclass correlation coefficient (ICC_2_, absolute agreement) and the coefficient of variation (CoV) for intra-lesion variability.

Two complementary statistical metrics were used to evaluate radiomic feature stability across imaging methods:

1. Coefficient of variation (CoV)
  The CoV was computed for each feature within individual lesions to quantify intra-lesion variability. For each feature class, the median of CoV values were reported.
2. Intraclass Correlation Coefficient (ICC)
  Stability between imaging methods (non-PVC, RL, and RVC) was quantified using ICC(2,1) for absolute agreement for consistency, both derived from a two-way mixed-effects ANOVA model. Lesion identifiers were treated as random effects and imaging methods as fixed raters. Analytical 95% confidence intervals (CIs) were estimated using the F distribution. Following established conventions, ICC values were interpreted as: poor (< 0.5), moderate (0.5–0.75), good (0.75–0.9), and excellent (> 0.9) agreement.

Differences across imaging methods were examined using the Friedman test, with subsequent linear mixed-effects modeling to account for within-patient correlations (patient ID as a random effect). Multiple testing correction was performed using the Benjamini–Hochberg false discovery rate (FDR) procedure with a significance threshold of FDR < 0.05.

For comparisons across lesion volumes and tissue categories, pairwise Mann–Whitney U tests were applied to ICC(2,1) distributions. The ΔMedian ICC was defined as the difference between the median ICCs of two groups, and all *p*-values were adjusted for multiple comparisons using FDR correction.

The normality of the intraclass correlation coefficient (ICC_2_ estimate) distributions was assessed using the Shapiro–Wilk test for each lesion volume and tissue group. All groups showed significant deviations from normality (*p* < 1 × 10^−7^ across all comparisons), justifying the use of non-parametric Mann–Whitney U tests for pairwise group comparisons.

All data management and statistical analyses were performed in Python (v3.10) using the libraries Pingouin (v0.5.5), SciPy (v1.16.2), and StatsModels (v0.14.5), with numerical computations handled via NumPy (v2.0.2).

## Results

The study included 131 patients with a mean age of 40.8 ± 20.4 years, 70 (53.4%) male and 61 (46.6%) female. Regarding disease type, 73 (58.9%) had HL and 51 (41.1%) had NHL. A total of 1,603 lesions were analyzed, and 93 radiomic features were successfully extracted from both non-PVC and PVC-corrected PET images. stability was quantified using the intraclass correlation coefficient (ICC_2_) for absolute agreement and the coefficient of variation (CoV) for intra-lesion variability.

### Effect of lesion volume on feature stability

**Fig. 2:**
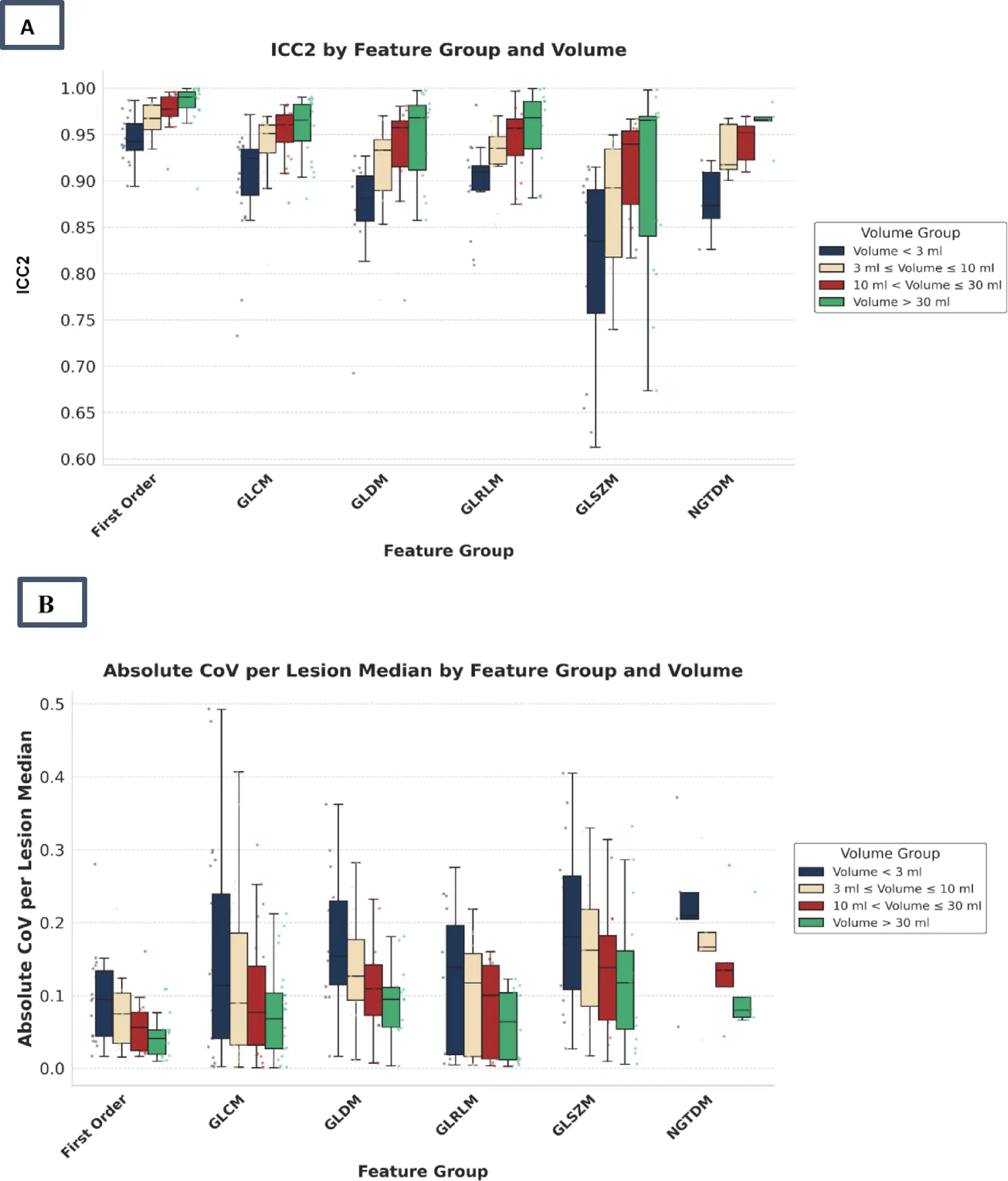
Radiomic feature stability across lesion volume groups. **A** ICC2 (absolute agreement) per feature group (First Order, GLCM, GLDM, GLRLM, GLSZM, NGTDM) for lesions of < 3 mL (dark blue), 3–10 mL (wheat), 10–30 mL (red), and > 30 mL (green). **B** Absolute median CoV per lesion for the same feature groups and volume categories. Boxplots show distributions, overlaid points represent individual values. Higher ICC2 and lower CoV indicate greater stability. High-resolution versions of these figures are available in Supplementary Fig. 2A, B

Figure 2A summarizes ICC_2_ across four lesion-volume groups (< 3 mL, 3–10 mL, 10–30 mL, and > 30 mL). Feature stability increased consistently with lesion size. Small lesions (< 3 mL) exhibited the lowest stability (median ICC_2_ = 0.84–0.94), whereas large lesions (> 30 mL) achieved excellent stability (median ICC_2_ > 0.95). Correspondingly, CoV values decreased with increasing volume (Fig. 2B), confirming reduced variability in larger lesions. First-Order and GLCM features were the most robust (CoV ≈ 0.04–0.10), while GLSZM and NGTDM features were highly variable in small lesions (CoV ≈ 0.17–0.21). Table 1 presents the pairwise Mann–Whitney U comparisons between lesion-volume groups. Lesions < 3 mL were significantly less reproducible than all larger groups (ΔMedian ICC_2_ = −0.035 to − 0.060, all FDR < 0.001), confirming that stability improves with increasing lesion size. Intermediate volumes (3–10 mL vs. 10–30 mL) also showed significant yet smaller differences.

### Effect of tissue type on stability

**Fig. 3:**
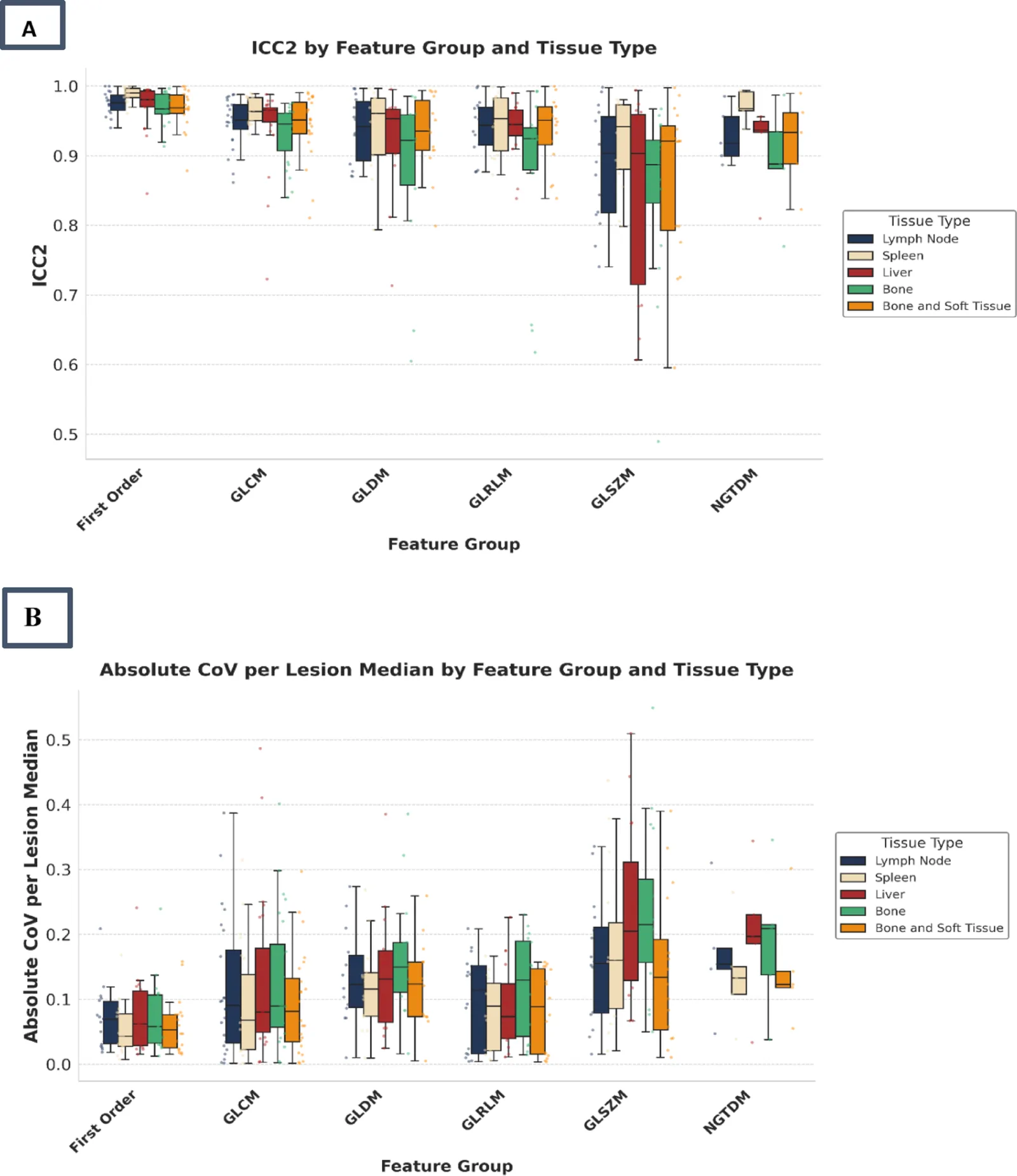
Radiomic feature stability across tissue types. **A** ICC2 (absolute agreement) per feature group (First Order, GLCM, GLDM, GLRLM, GLSZM, NGTDM) for different tissues: Lymph Node (Nodal), Spleen (Extranodal), Liver (Extranodal), Bone (Extranodal), Bone and Soft Tissue (Extranodal). **B** Absolute median CoV per lesion for the same feature groups and tissue types. Boxplots show distributions, overlaid points represent individual values. Higher ICC2 and lower CoV indicate greater stability. High-resolution versions of these figures are available in Supplementary Fig. 3A, B

Figures 2B and 3A display ICC_2_ and CoV distributions across five tissue categories (lymph node, spleen, liver, bone, and bone/soft tissue). Bone lesions demonstrated the highest stability (median ICC_2_ ≈ 0.95) with low CoV (≈ 0.08), whereas lymph node and spleen lesions showed moderate stability (ICC_2_ = 0.88–0.94). Liver and mixed bone–soft-tissue regions exhibited greater variability, particularly in texture-related features. Table 2 demonstrates that lymph node features were significantly less reproducible than spleen (ΔMedian ICC_2_ = −0.015, FDR = 0.0263) but slightly more stable than bone (ΔMedian ICC_2_ = 0.020, FDR = 0.0246). No significant differences were observed for liver or mixed tissues after FDR correction.

### Feature-class–specific stability

Table 3 summarizes ICC_2_ and CoV values across six feature classes. First-Order and GLCM features exhibited the highest robustness (ICC_2_ = 0.92–0.99, CoV = 0.04–0.11) across all lesion sizes and tissues. GLDM and GLRLM features showed moderate stability (ICC_2_ = 0.88–0.97) that improved with lesion size. Conversely, GLSZM and NGTDM features were highly sensitive to both lesion volume and tissue heterogeneity, showing poor stability in small or heterogeneous regions but reaching excellent agreement (ICC_2_ ≈ 0.96) in larger, homogeneous lesions.

### Heatmap-based visualization of stability patterns

**Fig. 4:**
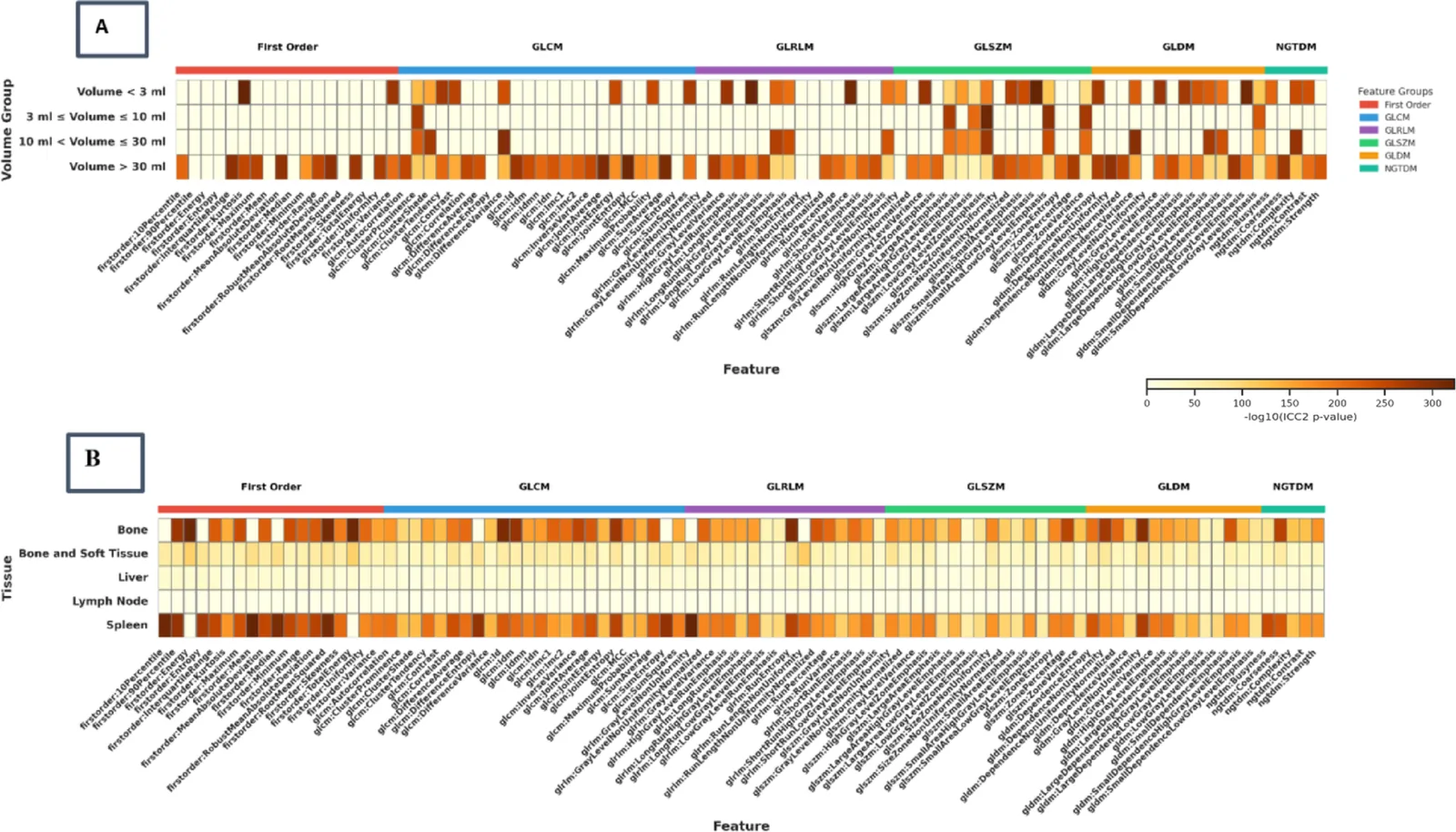
Radiomic feature stability visualized as heatmaps based on –log_10_(ICC_2_ *p*-values). **A** Statistical significance (– log_10_(p)) of ICC_2_ values across lesion volume groups (< 3 mL, 3–10 mL, 10–30 mL, and > 30 mL). **B** Statistical significance (– log_10_(p)) of ICC_2_ values across tissue categories (bone, bone and soft tissue, liver, lymph node, and spleen). Columns represent individual radiomic features grouped by category (First Order, GLCM, GLRLM, GLSZM, GLDM, NGTDM). Higher – log_10_(p) values (darker shades) indicate features with more statistically significant ICC_2_ stability. High-resolution versions of these figures are available in Supplementary Fig. 4A, B

**Fig. 5:**
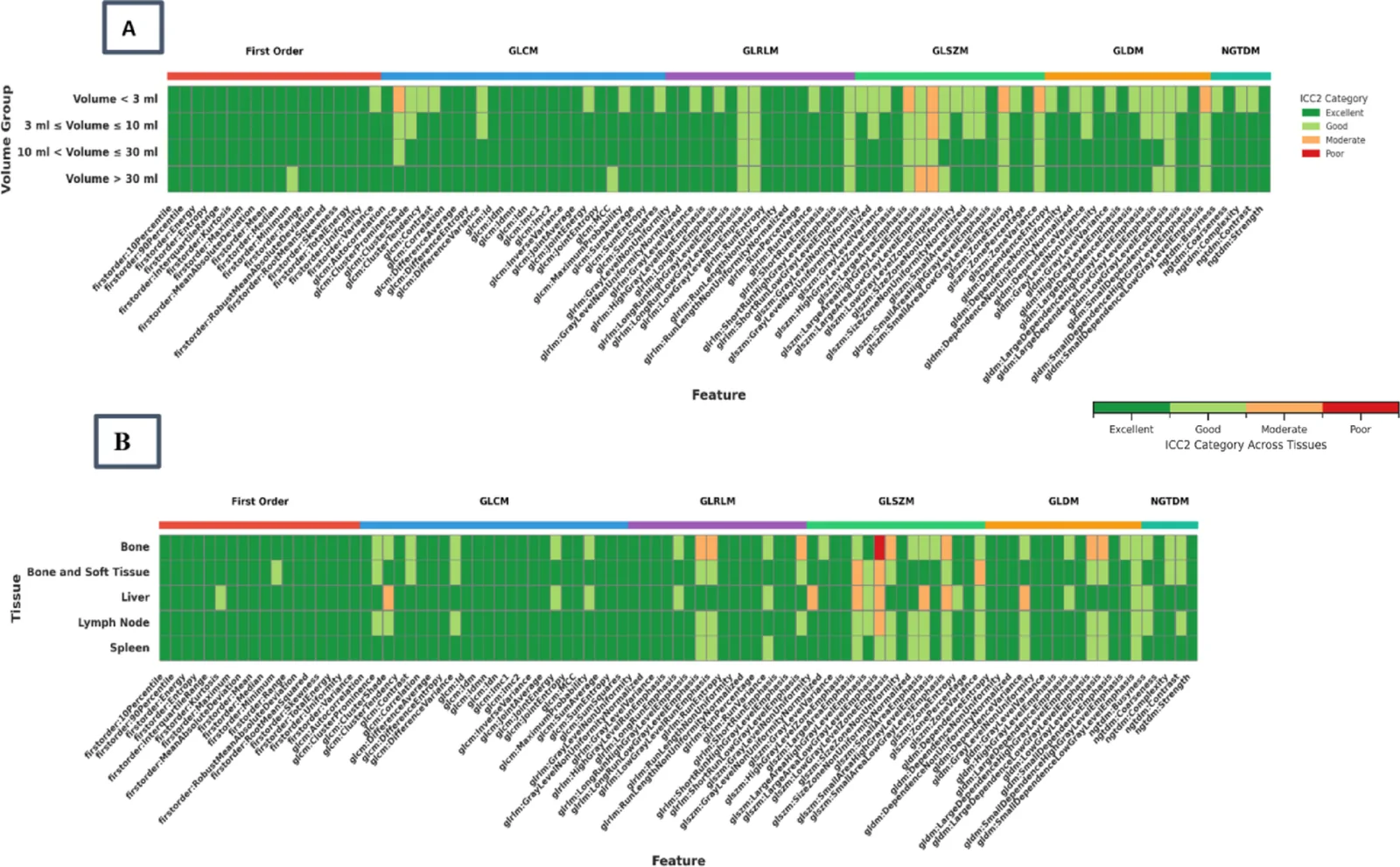
Categorical heatmap of radiomic feature stability (ICC_2_ categories) across lesion volume and tissue groups. Each cell represents the qualitative category of ICC_2_ stability: Excellent (≥ 0.90), Good (0.75–0.90), Moderate (0.50–0.75), and Poor (< 0.50). **A** ICC_2_ categories across four lesion volume groups (< 3 mL, 3–10 mL, 10–30 mL, and > 30 mL), illustrating the dependence of feature stability on lesion size. **B** ICC_2_ categories across tissue types (lymph node, spleen, liver, bone, and bone/soft tissue), showing inter-tissue differences in feature stability. Color bars indicate stability categories from Excellent (green) to Poor (red). High-resolution versions of these figures are available in Supplementary Fig. 5A, B

**Fig. 6:**
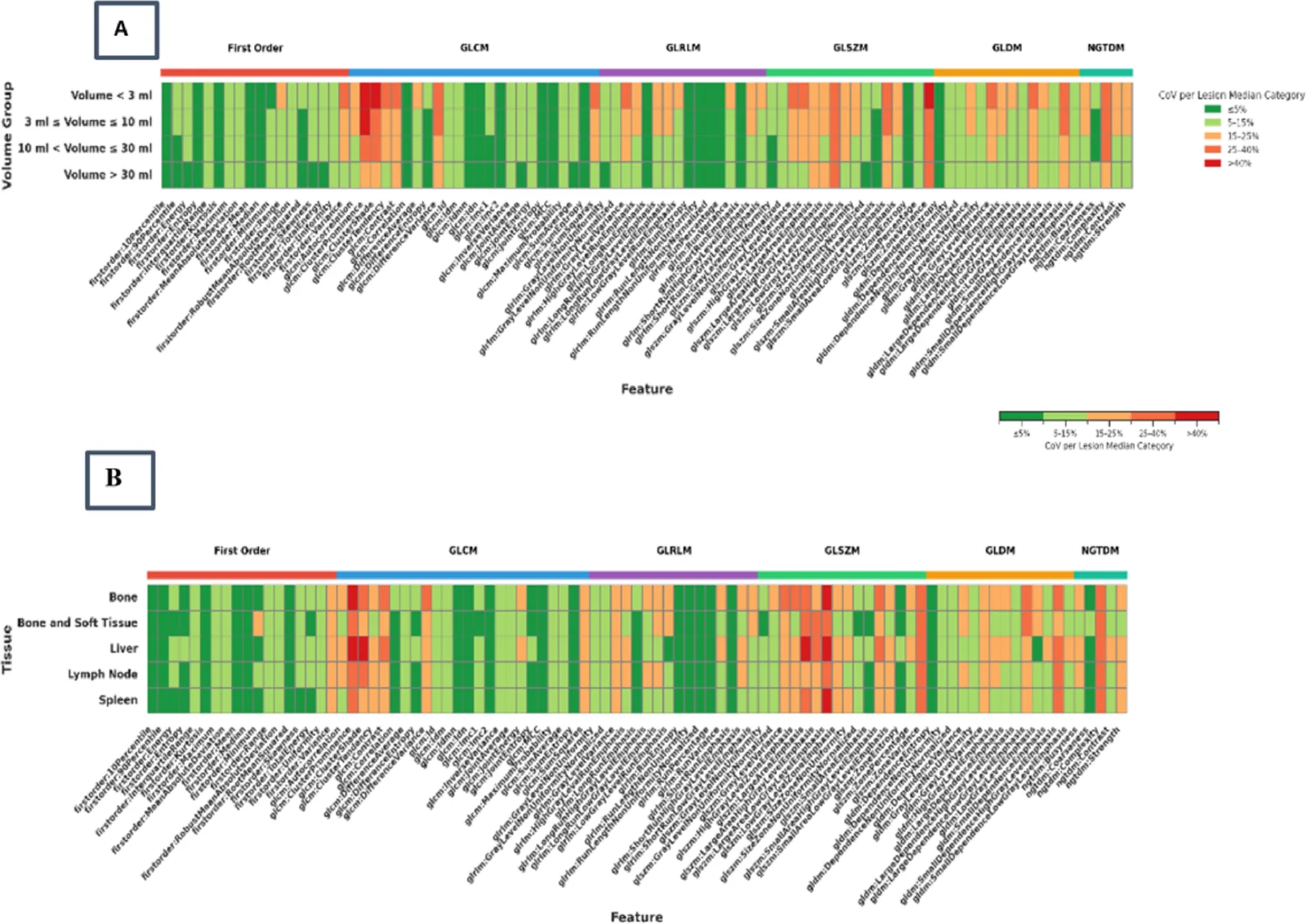
Categorical heatmap of absolute Coefficient of Variation (CoV) across lesion volume and tissue groups. Each cell represents the median absolute CoV category for a given radiomic feature: ≤ 5% (Excellent), 5–15% (Good), 15–25% (Moderate), 25–40% (High), and > 40% (Very High). **A** CoV categories across four lesion-volume groups (< 3 mL, 3–10 mL, 10–30 mL, > 30 mL), illustrating the effect of lesion size on measurement variability. **B** CoV categories across five tissue types (lymph node, spleen, liver, bone, and bone/soft tissue), demonstrating inter-tissue differences in radiomic feature stability. Color scales indicate relative variability, with greener shades corresponding to lower variability (higher stability) and redder shades to higher variability (lower stability). High-resolution versions of these figures are available in Supplementary Fig. 6A, B

Figure 4 visualizes radiomic stability significance as – log_10_(ICC_2_ *p*-values). Features in larger lesions and bone tissues exhibit darker shades, indicating higher statistical significance. Figure 5 presents categorical ICC_2_ stability maps across lesion volumes and tissues, showing that small lesions and heterogeneous regions (e.g., lymph node, spleen) have a higher proportion of moderate or poor categories, while larger and homogeneous lesions (e.g., bone) predominantly fall within the excellent range. Figure 6 shows categorical CoV heatmaps, where higher stability corresponds to lower CoV values (green shades). Variability increases notably in small lesions (< 3 mL) and complex tissues (liver, lymph node), especially in GLSZM and NGTDM feature classes.

Figures 2, 3, 4, 5, and 6 collectively demonstrate that both lesion volume and tissue type strongly influence radiomic stability. Small lesions and metabolically heterogeneous tissues (e.g., lymph node, spleen) show reduced stability, whereas larger and structurally uniform lesions (e.g., bone) exhibit consistent, high stability. Among all feature families, First-Order and GLCM metrics remain the most reliable, while GLSZM and NGTDM are highly sensitive to lesion size and tissue heterogeneity

### Summary of trends

**Table 1:** Pairwise comparison of lesion-volume groups based on ICC_2_ (Mann–Whitney U test)

Comparison	ΔMedian ICC2	*p*-value (Mann-Whitney)	FDR	Interpretation
Volume < 3 mL versus 3 mL ≤ Volume ≤ 10 mL	− 0.035	6.03E−07	0	Significant
Volume < 3 mL versus 10 mL < Volume ≤ 30 mL	− 0.049	1.31E−12	0	Significant
Volume < 3 mL versus Volume > 30 mL	− 0.06	5.12E−14	0	Significant
3 mL ≤ Volume ≤ 10 mL versus 10 mL < Volume ≤ 30 mL	− 0.014	0.00089	0.0011	Significant
3 mL ≤ Volume ≤ 10 mL versus Volume > 30 mL	− 0.024	2.18E−07	0	Significant
10 mL < Volume ≤ 30 mL versus Volume > 30 mL	− 0.011	0.00241	0.0024	Significant

ΔMedian ICC_2_ represents the difference in median ICC_2_ values between groups. FDR-adjusted *p*-values are reported; significant results (FDR < 0.05) are indicated. Smaller lesions (< 3 mL) show significantly lower stability than larger ones across all comparisons

**Table 2:** Pairwise comparison of tissue types based on ICC_2_ (Mann–Whitney U test)

Comparison	ΔMedian ICC2	*p*-value (Mann-Whitney)	FDR	Interpretation
Lymph Node versus Spleen	− 0.015	0.015807	0.0263	Significant
Lymph Node versus Liver	− 0.003	0.709021	0.7728	NS
Lymph Node versus Bone	0.02	0.010454	0.0246	Significant
Lymph Node versus Bone and Soft Tissue	0.003	0.591542	0.7394	NS
Spleen versus Liver	0.011	0.003721	0.0186	Significant
Spleen versus Bone	0.035	1.24E−06	0	Significant
Spleen versus Bone and Soft Tissue	0.018	0.006242	0.0208	Significant
Liver versus Bone	0.023	0.012306	0.0246	Significant
Liver versus Bone and Soft Tissue	0.007	0.772787	0.7728	NS
Bone versus Bone and Soft Tissue	− 0.017	0.037932	0.0542	NS

ΔMedian ICC_2_ represents the difference in median ICC_2_ values between tissue categories. FDR-adjusted *p*-values are reported; results with FDR < 0.05 are considered significant (“NS” = not significant). Lymph node features were slightly less reproducible than spleen but more stable than bone; bone and spleen generally exhibited the highest stability

**Table 3:** Median Coefficient of Variation (CoV) and Intraclass Correlation Coefficient (ICC_2_) of radiomic features across lesion-volume ranges

Feature category	Volume category	Number of lesions (*n*)	Median CoV	Median ICC
First order	Volume < 3 mL	337	0.094	0.942
First order	3 mL≤ Volume ≤ 10 mL	516	0.075	0.967
First order	10 mL < Volume ≤ 30 mL	315	0.056	0.978
First order	Volume > 30 mL	134	0.041	0.990
GLCM	Volume < 3 mL	337	0.114	0.924
GLCM	3 mL ≤ Volume ≤ 10 mL	516	0.090	0.951
GLCM	10 mL< Volume ≤ 30 mL	315	0.077	0.961
GLCM	Volume > 30 mL	134	0.068	0.965
GLDM	Volume < 3 mL	337	0.154	0.881
GLDM	3 mL ≤ Volume ≤ 10 mL	516	0.127	0.933
GLDM	10 mL < Volume ≤ 30 mL	315	0.109	0.958
GLDM	Volume > 30 mL	134	0.095	0.968
GLRM	Volume < 3 mL	337	0.139	0.910
GLRM	3 mL ≤ Volume ≤ 10 mL	516	0.117	0.935
GLRM	10 mL < Volume ≤ 30 mL	315	0.100	0.957
GLRM	Volume > 30 mL	134	0.064	0.968
GLSZM	Volume < 3 mL	337	0.175	0.835
GLSZM	3 mL≤ Volume ≤ 10 mL	516	0.162	0.892
GLSZM	10 mL < Volume ≤ 30 mL	315	0.139	0.940
GLSZM	Volume > 30 mL	134	0.117	0.965
NGTDM	Volume < 3 mL	337	0.209	0.873
NGTDM	3 mL ≤ Volume ≤ 10 mL	516	0.166	0.917
NGTDM	10 mL < Volume ≤ 30 mL	315	0.135	0.952
NGTDM	Volume > 30 mL	134	0.080	0.966

Median CoV and ICC_2_ values are reported for six feature categories (First Order, GLCM, GLDM, GLRLM, GLSZM, NGTDM) across four lesion-volume groups (< 3 mL, 3–10 mL, 10–30 mL, > 30 mL). “n” denotes the number of lesions in each category. stability (higher ICC_2_ / lower CoV) improves consistently with increasing lesion volume across all feature classes

## Discussion

This study shows that PVC using deconvolution-based algorithms, Richardson–Lucy (RL) and Reblurred Van Cittert (RVC), substantially improves the stability of ^18^F-FDG PET radiomic features in lymphoma across nodal and extranodal sites. Consistent with the hypothesis that PVC mitigates PVE, stability increased with lesion size and varied by tissue type. These findings are supported by the convergent patterns observed in Figs. 2, 3, 4, 5, and 6 and the statistics summarized in Tables 1, 2 and 3.

The present study demonstrates that lesion size plays a decisive role in determining radiomic feature stability after PVC. Larger lesions (> 30 ml) consistently exhibited excellent ICC_2_ values (0.965–0.990), whereas smaller ones (< 3 ml) showed moderate-to-high stability (0.835–0.942). This size-dependent pattern aligns with the known physical limitations of PET imaging, where PVE dominate in small objects because of the system’s finite spatial resolution (4.3–5.0 mm FWHM in the GE Discovery 690 used here) [31]. By compensating for PVE, PVC effectively restores spatial contrast and stabilizes textural metrics, particularly those sensitive to lesion size and heterogeneity [19, 32]. The significant ICC_2_ differences across lesion-size groups (ΔMedian ICC_2_ = − 0.035 to − 0.060, FDR < 0.001; Table 1) thus reaffirm that lesion volume remains a primary determinant of radiomic stability even after correction.

While the improvement in stability is evident, the uniformly high ICC_2_ values (> 0.90 across most categories; Table 3) suggest that methodological factors may also contribute. Features with inherently low sensitivity to intensity variations, such as First Order statistics (e.g., firstorder:10Percentile), achieved ICC_2_ up to 0.990 with CoV as low as 0.041, whereas complex textural matrices (GLSZM, NGTDM) exhibited reduced stability in smaller lesions but still reached ICC_2_ ≥ 0.965 for large volumes (Table 3; Fig. 6). Likewise, in brain PET research, RL and RVC achieved ICC ≥ 0.75 for most features, with only highly homogeneous regions such as the cerebellum approaching ICC ≥ 0.9 [18]. Hence, the high ICC_2_ values frequently exceeding 0.95 observed in our large lesions likely reflect both effective PVE correction and the high standardization of our imaging workflow (single scanner, VPHDS reconstruction, and standardized quantitative imaging practices [33], with IBSI-compliant discretization [28]), yet could also indicate a ceiling effect or limited variability between the RL and RVC algorithms.

Recent brain PET radiomics studies evaluating multiple PVC methods (including RL and RVC) reported ICC values ranging from < 0.5 in heterogeneous regions (e.g., fusiform gyrus, brainstem) to ≥ 0.9 in homogeneous regions (e.g., cerebellum, lingual gyrus), with over 60% of features exhibiting COV < 25% [17]. Similarly, first-order features showed high stability (e.g., FO-minimum with COV ≤ 5% in 100% of cases), while higher-order texture features often had large COV (> 20%) in 80–95% of cases, and ~ 80% of features in the amygdala had COV < 10% [34].In contrast, the present lymphoma study achieved generally higher ICC_2_ values (median > 0.95 for lesions > 30 mL, particularly in uniform tissues such as bone and spleen), likely due to larger lesion volumes, greater structural homogeneity, and the highly standardized single-center imaging protocol. Notably, the generally high ICC_2_ values (exceeding 0.90 for most features) in our dataset compare favorably with values typically reported in the literature [18], which may reflect reduced segmentation variability from expert-supervised semi-automatic delineation [27] and the controlled single-scanner setup [9, 12].

Tissue-specific analyses further demonstrate how biological heterogeneity affects feature robustness. Bone and spleen lesions displayed the highest stability (median ICC_2_ ≈ 0.95), while lymph nodes and liver showed lower stability (0.88–0.95; CoV 10.3–13.4%) (Fig. 5; Table 2). This gradient mirrors previous evidence from brain PET radiomics studies that homogeneous regions (e.g., cerebellum and lingual gyrus) yield higher stability (ICC ≥ 0.9), whereas heterogeneous regions (e.g., fusiform gyrus and brainstem) show poorer stability (ICC < 0.5) [18] .Within lymphoma, this distinction is clinically meaningful: extranodal sites like spleen and liver often exhibit diffuse uptake patterns, which likely explain the lower stability of GLDM and GLRLM features observed in these organs. High ICC_2_ values may partly reflect methodological factors, as single-center design and similar RL/RVC algorithms can inflate stability. Broader validation with segmentation-based PVC pipelines [35] and deep-learning PVC methods [36, 37] is needed to confirm robustness. In lymphoma, baseline ^18^F-FDG PET radiomics predicts outcomes [7, 38]; our results show PVC improves feature stability, especially in large lesions where PVE, not motion as in NSCLC [17], is dominant.

**Fig. 7:**
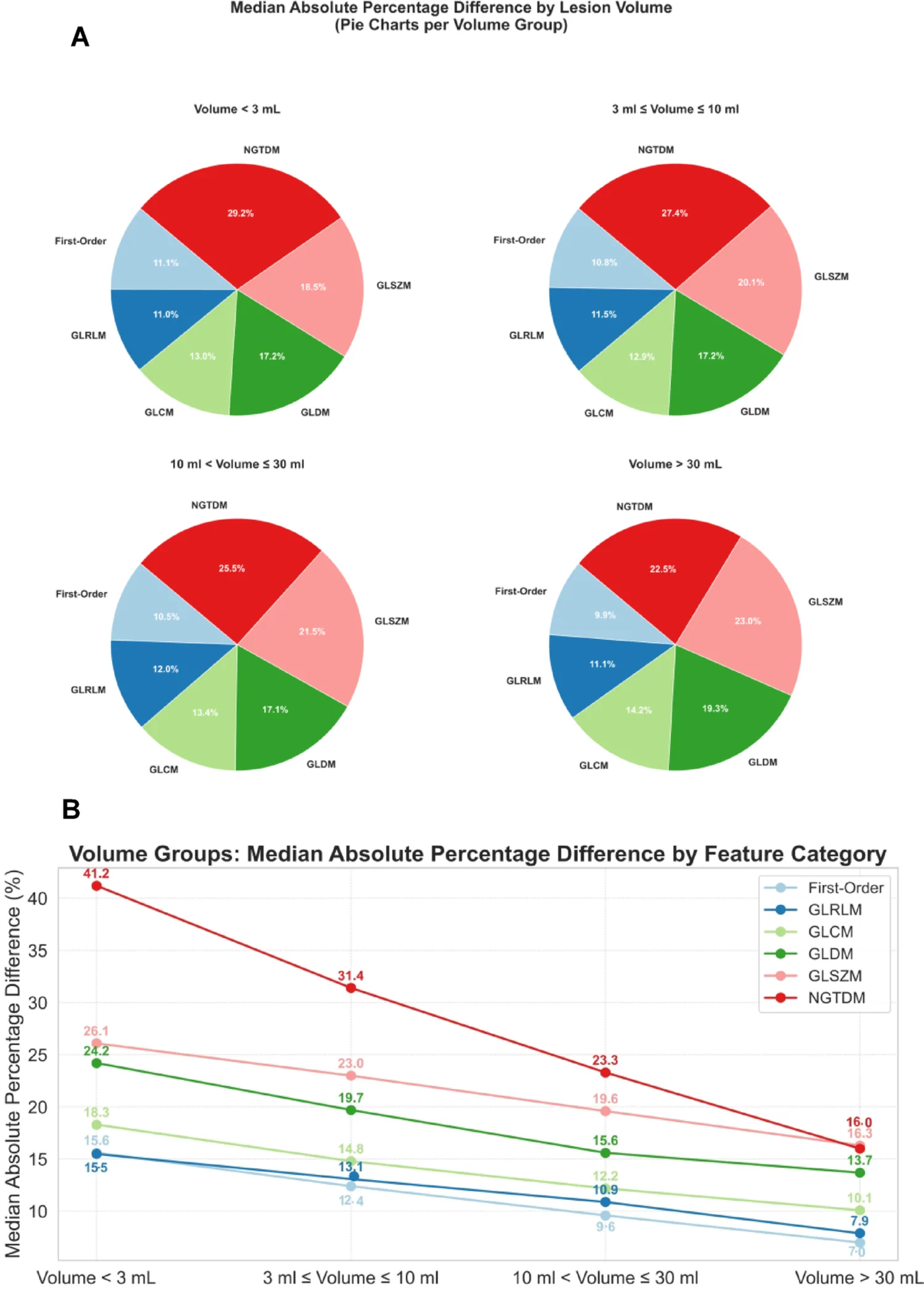
Median Absolute Percentage Difference of radiomic features across lesion volumes. **A** Pie charts per volume group. **B** Line charts showing feature trends, highlighting the most and least robust features. High-resolution versions of these figures are available in Supplementary Fig. 7A, B

**Fig. 8:**
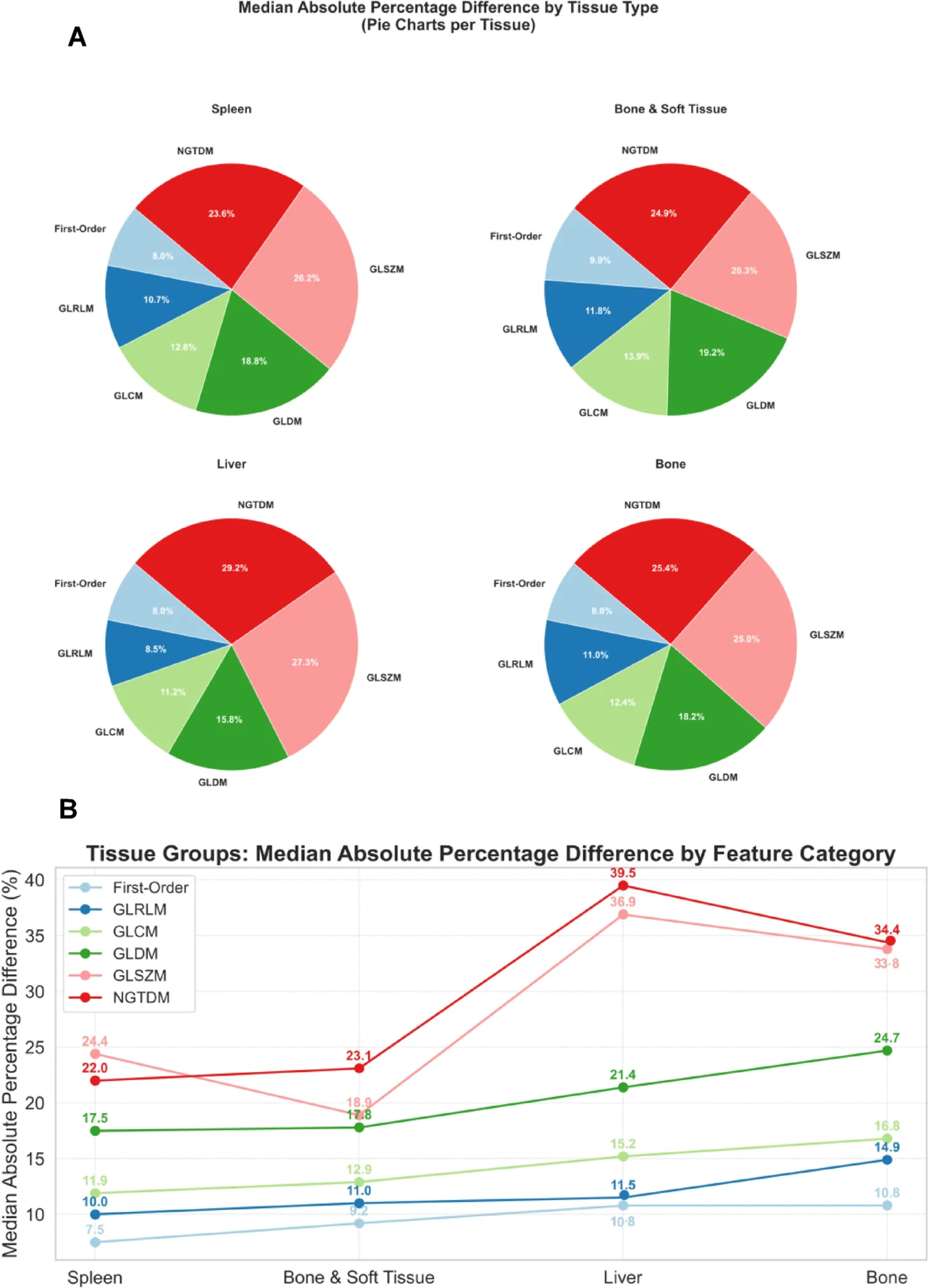
Median Absolute Percentage Difference of radiomic features across tissue types. **A** Pie charts per tissue group. **B** Line charts showing feature trends, highlighting the most and least robust features. High-resolution versions of these figures are available in Supplementary Fig. 8A, B

To provide a direct quantitative assessment of PVC’s impact on radiomic feature values, we calculated the median absolute percentage difference between non-PVC and PVC-corrected images (RL and RVC algorithms) for each feature category, stratified by lesion volume and tissue type (Table 4; Figs. 7 and 8).

**Table 4:** Median absolute percentage differences in radiomic feature values between non-PVC and PVC-corrected images (RL and RVC algorithms), stratified by lesion volume and tissue type

Groups	Feature category	NON PVC versus RL (%)	NON PVC versus RVC (%)	Average NON PVC versus PVC (%)
Volume < 3 mL	First-Order	16	15	15.6
Volume < 3 mL	GLRLM	15.8	15.2	15.5
Volume < 3 mL	GLCM	19	17.8	18.3
Volume < 3 mL	GLDM	25.2	23.9	24.2
Volume < 3 mL	GLSZM	26.8	26	26.1
Volume < 3 mL	NGTDM	42.3	39.9	41.2
Volume < 3 mL	Overall median	20.6	19.4	19.9
3 mL ≤ Volume ≤ 10 mL	First-order	12.7	12	12.4
3 mL≤ Volume ≤ 10 mL	GLRLM	13.4	12.9	13.1
3 mL≤ Volume ≤ 10 mL	GLCM	15.2	14.6	14.8
3 mL ≤ Volume ≤ 10 mL	GLDM	20.6	19.6	19.7
3 mL ≤ Volume ≤ 10 mL	GLSZM	23.8	22.3	23
3 mL≤ Volume ≤ 10 mL	NGTDM	31.5	30.4	31.4
3 mL≤ Volume ≤ 10 mL	Overall median	16.6	15.9	16.2
10 mL< Volume ≤ 30 mL	First-order	9.8	9.5	9.6
10 mL< Volume ≤ 30 mL	GLRLM	11.1	11	10.9
10 mL< Volume ≤ 30 mL	GLCM	12.5	12	12.2
10 mL < Volume ≤ 30 mL	GLDM	16	15.9	15.6
10 mL < Volume ≤ 30 mL	GLSZM	20	19.9	19.6
10 mL < Volume ≤ 30 mL	NGTDM	23.7	23.3	23.3
10 mL < Volume ≤ 30 mL	Overall median	13.4	13	13.1
Volume > 30 mL	First-order	7	7	7
Volume > 30 mL	GLRLM	8.1	7.9	7.9
Volume > 30 mL	GLCM	10.3	9.9	10.1
Volume > 30 mL	GLDM	14	13.5	13.7
Volume > 30 mL	GLSZM	16.9	16.6	16.3
Volume > 30 mL	NGTDM	16.3	15.2	16
Volume > 30 mL	Overall median	10.5	10.2	10.3
Bone	First-order	11	10.4	10.8
Bone	GLRLM	15.4	14.8	14.9
Bone	GLCM	17.3	16.3	16.8
Bone	GLDM	26.1	23.9	24.7
Bone	GLSZM	35.4	32.5	33.8
Bone	NGTDM	36.4	32.8	34.4
Bone	Overall median	19.7	18.3	18.9
Spleen	First-order	7.7	7.4	7.5
Spleen	GLRLM	10.2	9.9	10
Spleen	GLCM	12.3	11.5	11.9
Spleen	GLDM	17.3	17.7	17.5
Spleen	GLSZM	25.7	23.9	24.4
Spleen	NGTDM	23	22.3	22
Spleen	Overall median	13.2	12.5	12.9
Liver	First-order	11.4	10.4	10.8
Liver	GLRLM	11.2	10.5	11.5
Liver	GLCM	15.3	14.5	15.2
Liver	GLDM	20.5	21.4	21.4
Liver	GLSZM	36.2	37.6	36.9
Liver	NGTDM	42.3	33.6	39.5
Liver	Overall median	17	16.1	16.6
Bone & Soft Tissue	First-Order	9.4	9.2	9.2
Bone & Soft Tissue	GLRLM	10.8	11	11
Bone & Soft Tissue	GLCM	12.9	12.5	12.9
Bone & Soft Tissue	GLDM	17.6	17.4	17.8
Bone & Soft Tissue	GLSZM	18.6	19.7	18.9
Bone & Soft Tissue	NGTDM	24.4	22.2	23.1
Bone & Soft Tissue	Overall median	13.3	13	13.2

Across lesion volumes, PVC induced progressively smaller changes with increasing size, with overall median differences decreasing from 19.9% in small lesions (< 3 mL) to 10.3% in large lesions (> 30 mL). First-Order features were the most stable (median changes 7.0–15.6%), while higher-order texture features were more sensitive to PVC, particularly NGTDM (16.0–41.2%) and GLSZM (16.3–26.1%). This volume-dependent pattern aligns with the greater influence of partial volume effects in smaller lesions, where PVC substantially recovers lost signal and texture information.

Similarly, tissue-specific analysis revealed variation in PVC impact, with overall median changes ranging from 12.9% in spleen to 18.9% in bone lesions. Again, First-Order features exhibited the lowest differences (7.5–10.8%), whereas NGTDM and GLSZM showed the highest (up to 39.5% in liver and 34.4% in bone). These findings indicate that PVC has a more pronounced effect on texture metrics in metabolically heterogeneous or structurally complex tissues (e.g., liver, bone), while intensity-based features remain relatively robust across sites.

Collectively, these numerical effect sizes demonstrate PVC’s practical benefit in reducing feature variability caused by partial volume effects, particularly for texture-based radiomics in larger and more homogeneous lesions, supporting its integration into standardized PET radiomics workflows.

While our results demonstrate that deconvolution-based PVC (RL and RVC) can markedly enhance radiomic stability, they also underscore a broader methodological challenge in PET standardization. As highlighted by Cysouw et al. [19], the clinical translation of PVC remains controversial, although PVC improves quantitative accuracy in small lesions, its impact on diagnostic or prognostic performance across studies has been inconsistent. That meta-analysis revealed increased sensitivity but variable specificity after PVC, suggesting that the benefits depend strongly on lesion size, reconstruction protocol, and algorithm implementation rather than a universal improvement. Our findings extend this discussion to the radiomic domain: while PVC reduces variability and improves ICC_2_ metrics, such improvements may partly stem from the controlled conditions of a single-center design and limited methodological diversity. Therefore, stability alone cannot be equated with generalizability. Broader validation, incorporating multicenter data, motion-affected lesions, and newer deep-learning-based PVC frameworks, is essential to determine whether these gains translate into true robustness. Establishing standardized, reproducible PVC protocols across vendors and reconstruction pipelines remains a prerequisite for integrating PVC-corrected radiomic biomarkers into clinical decision-making.

Our study provides several novel contributions to PET radiomics. First, the large sample size enhances the statistical stability and generalizability of our findings. Second, unlike most prior studies that evaluate a single PVC method, we compared two widely used PVC algorithms, allowing for a more robust assessment of feature stability. Third, we observed that bone and spleen lesions exhibit higher robustness than lymph node or liver lesions, a finding with important clinical implications in lymphoma, where multi-organ involvement is common. Finally, our results offer practical guidance on whether PVC should be applied in PET radiomics studies and which feature classes remain trustworthy after correction, supporting harmonization efforts in multicenter trials.

Despite the promising stability gains achieved through deconvolution-based PVC, several limitations should be acknowledged. First, this was a single-center study conducted using a single PET/CT system (GE Discovery 690) with uniform reconstruction parameters. While this homogeneity minimizes technical variability, it may artificially elevate stability estimates and limit generalizability to multi-scanner or multi-vendor settings. Second, semi-automated segmentation, although refined by expert correction, introduces potential operator bias and reduces the independence of measurements, particularly in heterogeneous extranodal lesions. Third, only two classical iterative PVC algorithms (Richardson–Lucy and Reblurred Van Cittert) were evaluated; newer deep-learning-based PVC methods may further improve correction performance and should be compared systematically in future work. Fourth, we did not assess test–retest repeatability or multicenter harmonization, both of which are critical for validating radiomic biomarkers. Finally, as radiomic features were extracted under standardized conditions, further studies are needed to evaluate stability under realistic clinical variability, including differences in acquisition protocols, motion effects, and reconstruction settings. Additionally, although all segmentations were performed and manually refined by a single experienced nuclear medicine physician (with verification of a subset by a second reader), systematic interobserver stability testing was not conducted due to the large number of lesions (*n* = 1,603). Potential interobserver variability in manual refinements therefore remains a limitation. Addressing these limitations through cross-center validation and inclusion of modern data-driven PVC frameworks will be essential for establishing reproducible and standardized PET radiomics pipelines.

## Conclusion

In conclusion, partial volume correction substantially improves the stability of ^18^F-FDG PET radiomic features in lymphoma. Larger and more homogeneous lesions demonstrated the highest stability, confirming that mitigating partial volume effects is essential for reliable radiomic quantification. Among all feature classes, First-Order and GLCM features showed the greatest robustness, supporting their suitability for prognostic and response-assessment models. Direct quantification of feature value changes further highlights PVC’s benefit in reducing variability, especially for texture-based features in smaller or heterogeneous lesions. While the strong stability observed highlights the potential of PVC for standardizing PET radiomics, further multicenter and multi-method validation is required to ensure these improvements are consistent across scanners, protocols, and clinical settings.

## Supplementary Information

Below is the link to the electronic supplementary material.

Supplementary Material 1

## Data Availability

No datasets were generated or analysed during the current study.
